# Prediction of unplanned 30-day readmission for ICU patients with heart failure

**DOI:** 10.1186/s12911-022-01857-y

**Published:** 2022-05-02

**Authors:** M. Pishgar, J. Theis, M. Del Rios, A. Ardati, H. Anahideh, H. Darabi

**Affiliations:** 1 Department of Mechanical and Industrial Engineering, University of Illinois at Chicago, 842 W Taylor Street, MC 251, Chicago, IL 60607 USA; 2 Department of Emergency Medicine, Roy J. and Lucille A. Carver College of Medicine, University of Iowa, Iowa City, USA; 3 Department of Cardiology Medicine, University of Illinois at Chicago, Chicago, USA

**Keywords:** Hospital readmission, Heart failure, Process mining, Deep learning

## Abstract

**Background:**

Intensive Care Unit (ICU) readmissions in patients with heart failure (HF) result in a significant risk of death and financial burden for patients and healthcare systems. Prediction of at-risk patients for readmission allows for targeted interventions that reduce morbidity and mortality.

**Methods and results:**

We presented a process mining/deep learning approach for the prediction of unplanned 30-day readmission of ICU patients with HF. A patient’s health records can be understood as a sequence of observations called event logs; used to discover a process model. Time information was extracted using the DREAM (Decay Replay Mining) algorithm. Demographic information and severity scores upon admission were then combined with the time information and fed to a neural network (NN) model to further enhance the prediction efficiency. Additionally, several machine learning (ML) algorithms were developed to be used as the baseline models for the comparison of the results.

**Results:**

By using the Medical Information Mart for Intensive Care III (MIMIC-III) dataset of 3411 ICU patients with HF, our proposed model yielded an area under the receiver operating characteristics (AUROC) of 0.930, 95% confidence interval of [0.898–0.960], the precision of 0.886, sensitivity of 0.805, accuracy of 0.841, and F-score of 0.800 which were far better than the results of the best baseline model and the existing literature.

**Conclusions:**

The proposed approach was capable of modeling the time-related variables and incorporating the medical history of patients from prior hospital visits for prediction. Thus, our approach significantly improved the outcome prediction compared to that of other ML-based models and health calculators.

## Background

The prevalence of Heart Failure (HF) rises over time. Approximately, 6 million American adults (age > 20) had HF between 2015 to 2018 [1]. Despite the progress made in HF therapeutics, readmission rates remain high at nearly 20% [1–3]. Excessive unplanned readmissions and subsequent waste of medical resources have had financial implications that directly affect the overall performance of the hospitals. The Hospital Readmissions Reduction Program was established by the Affordable Care Act (ACA) in 2010 to encourage hospitals to avoid readmissions by penalizing the hospitals that exceed the expected thresholds [4]. Since 2012, hospitals have been penalized over $2.5 billion by the Centers for Medicare & Medicaid Services (CMS) for exceeding the unplanned 30-day readmission rates [5]. Unplanned ICU readmission may impose a severe financial burden on both hospitals and patients [6]. Readmissions were found to be associated with increased morbidity and mortality. The mortality rate of unplanned ICU readmission ranged between 26 and 58% [7]. The ICU readmission rate had increased over time rising from 4.6% in 1989 to 6.4% in 2003 [1]. Approximately 16% of unplanned ICU readmissions occurred within 30-days of initial hospital discharge [8, 9].

The Electronic Health Record (EHR) has been revolutionizing the health care decision-making processes through collecting and preserving medical data in a digital format. The use of the EHR has been allowing hospital systems to make intelligent data-informed decisions to address a wide range of problems from learning personalized prescriptions to maximizing the performance of hospitals [10, 11].

Artificial Neural Networks (ANNs), [12], is a popular network-based ML technique to address complex problems in various application domains [13–15]. Neural networks (NN) can be hardware-based (physical components represent neurons) or software-based (computer models), and they can employ a wide range of topologies and learning algorithms. NN involve feed-forward and back-propagation steps. The former model calculates the estimates for each observation in the training set, and in the latter, the errors are calculated to adjust the parameter estimates in the next iteration. NN generate their own features through a multi-layer structure of the network and use specific transformation of the input features called activation functions. Hence, NN are superior to many basic machine learning models in capturing underlying non-linearities when we have access to sufficient data. Many variants of NN have been proposed and developed for different settings; Convolutional Neural Networks (CNN) for image classifications and Recurrent Neural Networks (RNN) for time-series predictions [16].

Several ML and artificial intelligence techniques have been proposed to predict unplanned 30-day readmission of ICU patients with HF [11, 17]. However, the results of the developed models were not quite reliable.

Process mining analyzes and optimizes the sequence of events occurring during running processes, known as the event logs. The process mining approach has been used to enhance the healthcare processes [18]. However, the medical history of the patients from past hospital visits has not yet been used to predict unplanned readmission [19–21].

The present study aimed to introduce a novel process mining/deep learning approach that incorporated the past medical history of the patients from prior hospital visits and the time information related to the variables (Time State Samples (TSS)) to predict unplanned 30-day readmissions of the ICU HF patients. A prediction model was developed in accordance with the Transparent Reporting of a multivariable prediction model for Individual Prognosis Or Diagnosis (TRIPOD) initiative guidelines [22, 23].

## Methods

### Data source and inclusion criteria

We used the Medical Information Mart for Intensive Care III (MIMIC-III) public database, which contained deidentified clinical data of the patients who were admitted to the Beth Israel Deaconess Medical Center in Boston, Massachusetts [24]. MIMIC-III contained 38,597 adult patients and 49,785 hospital admissions from 2001 to 2012. This database consisted of various tables such as admission information, demographics, caregiver information, lab values, charted observations, discharge summary notes, and diagnosis codes.

In order to identify HF patients, specific ICD-9 codes related to the HF diagnosis including 398.91, 402.01, 402.11, 402.91, 404.01, 404.03, 404.11, 404.13, 404.91, 404.93, 428. XX were used in this study. Patients were included if any of these ICD9 codes appeared in the most recent admission following the standard approach in the existing literature [25]. Note that, patients who were diagnosed with HF in previous admissions, but were not diagnosed with HF in the current admission are not included. Among the patients who were diagnosed with HF in the current admission, we included those who had visited any hospital at least once before the current ICU hospital visit and had at least one future hospital admission of any type. The condition of having at least one hospital visit before the current admission is needed as the process mining approach requires an existing medical history as its input at the time prediction. Also, having at least one future admission of any type is to guarantee that the patient is still alive and refers to the same hospital system for his/her medical needs. Patients who died in the same hospital ICU or got discharged from a hospital ICU and died later in another hospital or other parts of the same hospital were excluded.

### Variable selection

Several variables were considered as the inputs to the model which are as follows: the admission type, the associated time of the admission, types of insurance, the discharge time, several lab measurements, various performed services, procedures, and diagnoses on the patients, and demographic information. The admission types were categorized either as planned or unplanned admission. The insurance group types were defined as Medicare, Medicaid, Private, Government, and Self-pay. Lab values typically obtained to predict HF were extracted for each patient including Blood Urea Nitrogen (BUN), Serum Creatinine, sodium ion, and pro-brain natriuretic peptide (NT-proBNP). The various types of performed services, procedures and diagnoses were considered in the form of CPT and ICD-9-CM codes. The demographic information including age, gender, and ethnicity was used as additional variables in the model.

### Conversion of EHR into event logs

The proper format of the input data for the process mining is event logs. Event logs contain the sequences of events as well as the associated time at which specific events occurred, which are referred to as timestamps.

The transformation of the EHR of the patients into the event logs was done based on the method reported by Theis et al. [21].

Thirteen different event types were defined in this section. Table 1 shows the mapping of each of the considered event types with the MIMIC-III tables.

**Table 1:** Mapping of the medical health records of the patients from the MIMIC-III database to the event logs

	Events
Admissions Patients	Admission type event Admission Insurance event Discharge event
Admissions Labevents D-Labitems	BUN Mean event Serum Creatinine Mean event NT_ proBNP Mean event Sodium-ion Mean event BUN std event Serum Creatinine std event NT_ proBNP std event Sodium-ion std event
Admissions Diagnoses_ICD	Elixhauser Comorbidity Score events
Diagnoses_ICD Procedures_ICD Cptevents	30 Artificial event abstractions

BUN, blood urea nitrogen; Pro-BNP, Pro-brain natriuretic peptide; std, standard deviation

### Event types and the associated timestamps

For each patient, we converted the EHR to events with the following sequence:

First, we considered the admission type event with the admission time as its timestamp. Admission type event was important since it distinguished whether the admission was planned or unplanned. The second event was the insurance type event with a timestamp of 1 ms after the timestamp of the admission type to maintain the order of the events. Insurance type was chosen since it could possibly affect the discharge/transfer rate.

The next set of events was the lab measurements. Specific HF-related lab measurements were chosen based on the literature [11, 17] and experts’ opinions. The lab measurements might be measured once or several times for each patient. Two types of events were created for each lab item, out of which one was the Mean of the specific lab item and the other was the Standard Deviation (std) of each lab item.

In the cases in which the lab item was measured once, the timestamp of the Mean event was set to the timestamp at which the lab item was measured initially. The timestamp for the std event was set to 1 ms after the timestamp of the Mean event. For the lab measurements, which were measured several times, we performed similarly for the Mean event. However, for the std event of such cases, the timestamp was set to the last time at which the lab item was measured.

We considered a separate set of events representing the comorbidities. Elixhauser comorbidity score was calculated by using the ICD-9 diagnosis codes for each hospital visit [26]. A specific comorbidity group can be determined by assigning points through the Elixhauser comorbidity score if particular ICD-9 codes are present. These comorbidity events were created because they represent what diseases have been diagnosed, whether the diseases are chronic, and the criticality of the patients. Additionally, based on the literature, these events were strongly associated with ICU readmission risk [27]. In cases where a point was assigned to a specific group, an event was created with the same name as in the comorbidity group. Since the focus of this paper was on readmission prediction, we needed specific event logs that separated all the timestamps of the events from the discharge timestamp (which was the final event). Therefore, the timestamps for these events were set to be very close to the discharge time of the relevant hospital visit. In cases where multiple comorbidity events were created, the timestamp for the second comorbidity event was set 1 ms after the timestamp of the first event. The same logic was applied for the next comorbidity events as well.

The artificial events were created from the sequence of CPT, and ICD-9-CM observations codes as by Theis et al. [21]. We considered these events since they represented the diagnoses and procedures of a patient which were likely to be important factors to predict readmission. The artificial events’ timestamps were set to the timestamps of the sequence of the observations plus 1 ms. These timestamps accordingly were compared with timestamps of the discharge event, and they were set to a time before the discharge timestamp to ensure the orders of the events were maintained.

In the end, the discharge event was created for each hospital admission and the timestamp of this event was set to the discharge time of the patient for the corresponding admission. This event was created since this was a point at which the next event (admission type event) would be predicted by using Decay Replay Mining (DREAM) algorithm [28].

Note that the addition of 1 ms to the timestamps in our conversion did not alter any information since the time dimension in MIMIC-III was days and subsequently negligible in our analysis.

### Label setup

A column was created to label the patients. If a HF patient was readmitted unplanned within 30-day, the label was set to TRUE. Otherwise, the label was set to FALSE. Moreover, a column was created to indicate the point at which the TSS information needs to be collected to prevent the data leakage. The collection of the TSS information was set at the discharge event, which means no time information regarding the future admission was collected by the DREAM algorithm.

### Unplanned 30-day readmission prediction

**Fig. 1:**
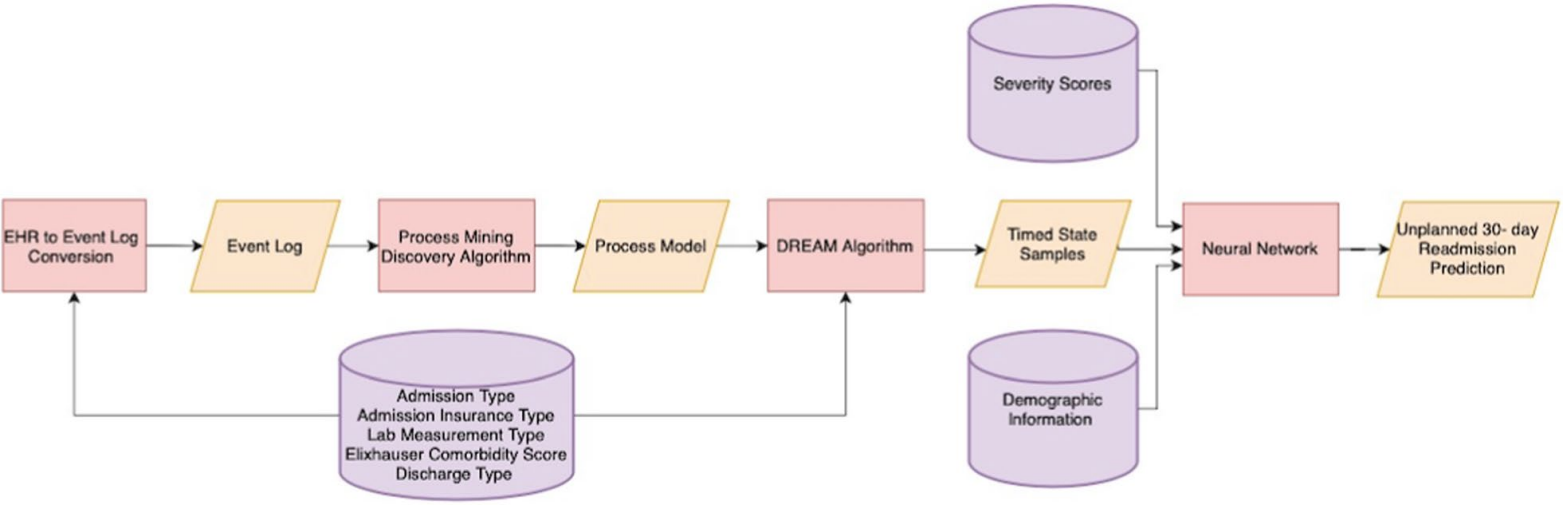
Overview of the methodology. This Figure illustrates the overview of the methodology. The admission, insurance, lab measurements, Elixhauser comorbidity, and the discharge information of the patients were extracted from MIMIC-III database and converted to an event log. The resultant event log was used as an input to the process mining discovery algorithm to produce a process model. The resultant process model along with the event logs were then fed to the DREAM algorithm and resulted in some time information related to the variables. The time information with the demographic and the severity scores of the patients were then fed to a NN to predict unplanned 30-day readmission of the ICU HF patients

**Fig. 2:**
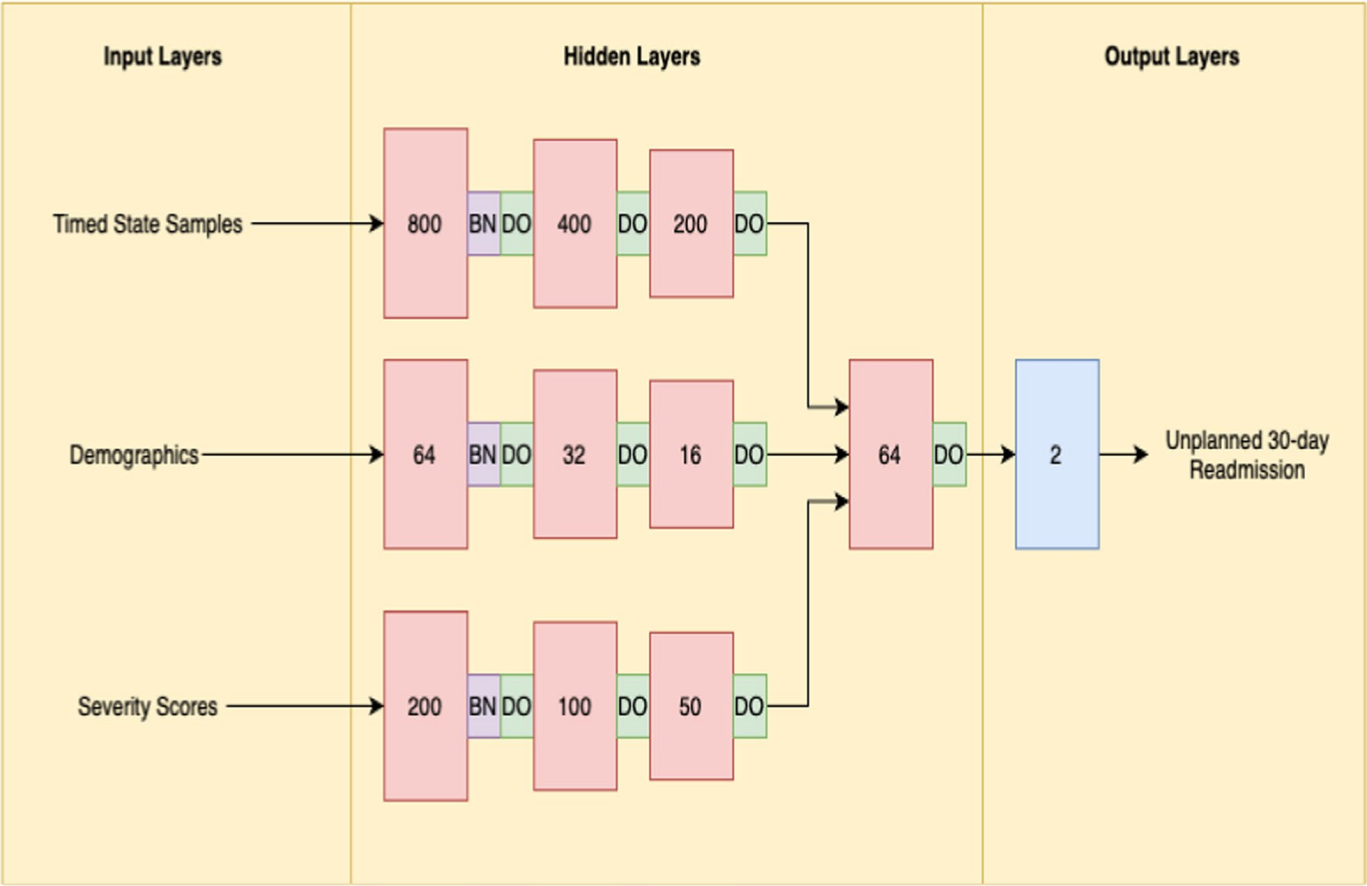
Architecture of Neural Network (NN). This Figure shows the details of the NN architecture. The timed state samples, demographics information, and the severity scores were fed separately to three branches which each branch contains three hidden layers. A batch normalization layer was added after the first hidden layer of each branch. Also, a dropout with a rate of 20% was used after the first, second, and third hidden layers. At the end, the output layer included softmax activation function to predict unplanned 30-day readmission of ICU HF patients

We proposed a process mining/deep learning approach for unplanned 30-day readmission prediction which is shown in Fig. 1. The resultant event logs were fed to the process mining discovery algorithm to produce a process model. The resultant process model along with the event logs were fed to the DREAM algorithm to generate the time information (TSS). The severity scores on admission day including the Charlson [29] and Elixhauser scores were used as variables. Charlson score method assigns higher weights to more severe and critical conditions as compared to Elixhauser that assigns the same weight to all conditions. To prevent data leakage, the severity scores were calculated based on the information that was available up to the discharge event of the current admission, and no information after the discharge event of the current admission was used for the calculation of the severity scores. Hence there is no data leakage involved. The generated TSS, together with the demographic information and the severity scores were then fed to a NN model to predict unplanned 30-day readmission of the ICU patients with HF. The architecture of the NN model is shown in Fig. 2 and is as follows: the time information, demographics information, and the severity scores were fed separately to three branches which each branch contains three hidden layers. These hidden layers were then concatenated and fed to a subsequent layer. For all hidden layers, a Rectified Linear Unit activation function was used to improve the performance of the model. Moreover, to improve the stability, a batch normalization layer was added after the first hidden layer of each branch [21]. Additionally, for regularization [21], a dropout with a rate of 20% was used after the first, second, and third hidden layers [21]. Moreover, the NN model was trained for 100 epochs using a batch size of 10. In the end, the output layer included a softmax activation function to predict unplanned 30-day readmission of ICU HF patients. Also, Adam optimizer was used as an optimizer function [30]. The corresponding source code is publicly available on our Github repository.

The proposed model was evaluated by calculating Area Under the Receiver Operating Characteristic curve (AUROC), precision, sensitivity, accuracy, and F-score on the test set. To obtain 95% Confidence Intervals (CIs) of the AUROC value, DeLong’s method was used [31].

### Baseline model development

To compare the results of the process mining/deep learning approach, several baseline models were utilized. These models were developed using ML algorithms. The baseline models were trained based on the MIMIC-III data cohorts to explore other ML algorithms for the prediction. The development of these models was utilized first by feeding the same possible variables as fed to the proposed model, but kept in the original tabular format, as opposed to the event logs format. The intuition behind this experiment was to compare the performance of the process mining approach, which consists of both the TSS information and the NN model, with that of the ML algorithms. Therefore, in this experiment we did not use the TSS information as an input to the ML algorithms. On the other hand, in another experiment, we compared the performance of the NN model with that of the ML algorithms. In this case, to make a fair comparison, we fed the TSS information to both the NN model and the ML algorithms. That means the ML algorithms and the NN model all received the same values of inputs, i.e. TSS, demographics, and severity scores. A variety of popular ML algorithms were evaluated to classify unplanned 30-day readmission of ICU patients with HF. These algorithms included Support Vector Machine [32], K-nearest neighbors [33], Decision Trees [34], Random Forest [35], XGBoost [36], and CatBoost [37]. The training process of these models included a grid search of model parameters. This search process aimed to find the best model which was determined based on the AUROC of the validation cohort.

### Statistical analysis between cohorts

The train and validation cohorts were compared using Chi-Square and two-sided t-tests. For the comparison of the categorical variables, Chi-Square tests were performed, and for continuous variables, t-tests were implied. The significant level was determined based on *P* < 0.05. Descriptive statistics, model development, and statistical analysis were conducted using Python, version 3.6.

### Variables impacts and ablation study

Shapley value analysis [38] was conducted on the test set to find out the impact of each variable in our proposed model prediction and to figure out which variable was particularly associated with readmissions. The Shapley values described the Mean contribution of each variable to the outcome across different coalitions [21]. Moreover, a variable ablation study was conducted on demographic information, severity scores, and event type in the event log to find out how the results change by removing specific variables. Furthermore, a layer-wise ablation study on the NN was done to demonstrate the architecture of the proposed NN was optimized.

## Results

### Cohort characteristics model completion

Following the approach for selection of HF patients discussed before in this paper, a subset of 3411 patients was selected from the MIMIC-III database. The selected cohort was then split into train/validation/ and test cohorts randomly with a ratio of 73.1/12.9/ and 14, which yielded a result of 2422 patients for train, 434 patients for validation, and 555 patients for the test cohorts. Moreover, the train and validation cohorts were used to discover the process model and the NN training. Furthermore, the best model was chosen based on its AUROC performance on the validation set, the model which led to the highest value of AUROC by using the validation dataset were chosen as the best model. Moreover, it was used for further evaluation on the test cohort. The description of the train and validation cohorts is presented in Table 2. The readmission rate for train, validation, and test were 23.9, 23.5, and 23.4% respectively, out of 2422 patients in the training cohort, 581 of them were readmitted unplanned within 30-day, out of 434 patients in the validation cohort, 102 of them were readmitted, and out of 555 patients in the validation cohort, 130 of them were readmitted. In terms of age, the validation cohort (70.4 years) was slightly older than the training cohort (69.9 years) with a *P* of 0.228 which showed there were no significant differences between cohorts. In terms of gender, the training cohort (47.6%) contained slightly more females compared to the validation cohort (46.3%). The whole distribution of the race was not significantly different between the cohorts [*P* = 0.270], of which the details are shown in Table 2. The proportions of white patients in the train and validation cohorts were 75.8%, and 74.9% respectively. The lab measurements were not significantly different between cohorts except for Urea Nitrogen which was 0.017.

**Table 2:** Comparison of the variables including outcome, demographics, and laboratory findings between train and validation cohorts

Characteristics	Train cohort (N = 2422)	Validation cohort (N = 434)	*P*
*Outcome variable N, (%)*			
Readmission	581 (23.9)	102 (23.5)	0.270
*Demographics*			
Age mean (std)	69.9 (14.3)	70.4 (13.9)	0.228
Female (%)	47.6	46.3	1.00
*Race N (%) *			0.270
African American	291 (12.0)	51 (11.8)	
Hispanic	76 (3.10)	18 (4.15)	
Others, non-Hispanic	170 (7.00)	37 (8.53)	
White	1835 (75.8)	325 (74.9)	
Asian	50 (2.10)	3 (0.691)	
*Laboratory findings mean (std)*			
Sodium	138.6 (4.58)	138.5 (4.80)	0.835
Urea nitrogen	34.4 (24.0)	32.6 (22.3)	0.017
NT proBNP	0.187 (0.380)	0.181 (0.385)	0.613
Serum creatinine	0.002 (0.04955)	0.004 (0.062)	0.083

Pro-BNP, Pro-brain natriuretic peptide

### Evaluation metrics, proposed and baseline models performance

The summary of the results for both proposed and baseline models are shown in Table 3, 4 and 5. The proposed approach resulted in the following metrics, AUROC = 0.930, 95% CI = [0.898–0.960], precision = 0.886, sensitivity = 0.805, accuracy = 0.841, and F-score = 0.800.

**Table 3:** Summary of the results for proposed model on train, validation, and test sets

Models	AUROC	AUROC 95% CI	Precision	Sensitivity	Accuracy	F-score
Proposed model performance on test set	0.930	[0.898–0.960]	0.886	0.805	0.841	0.800
Proposed model performance on validation set	0.942	[0.908–0.990]	0.905	0.831	0.881	0.863
Proposed model performance on train set	0.971	[0.914–1.00]	0.926	0.843	0.901	0.901

**Table 4:** Summary of the results for the baseline models on test set using the same possible inputs as fed to the proposed model, but in the original tabular format

Models	AUROC	AUROC 95% CI	Precision	Sensitivity	Accuracy	F-score
RF	0.713	[0.691–0.761]	0.750	0.801	0.828	0.760
XGBoost	0.701	[0.685–0.756]	0.731	0.804	0.826	0.763
CatBoost	0.704	[0.674–0.759]	0.692	0.800	0.829	0.752
SVM	0.680	[0.657–0.712]	0.691	0.801	0.828	0.753
Decision tree	0689	[0.669–0.721]	0.724	0.691	0.688	0.710
KNN	0.696	[0.657–0.731]	0.725	0.802	0.815	0.751

RF, random forest; SVM, support vector machine; KNN, K-nearest neighbors

**Table 5:** Summary of the results for the baseline model on test set using the same inputs (TSS, demographics, and severity scores) as fed to the proposed model

Models	AUROC	AUROC 95% CI	Precision	Sensitivity	Accuracy	F-score
RF	0.841	[0.793–0.864]	0.820	0.803	0.830	0.771
XGBoost	0.832	[0.748–0.843]	0.812	0.792	0.813	0.773
CatBoost	0.830	[0.779–0.859]	0.780	0.801	0.829	0.763
SVM	0.801	[0.763–0.843]	0.775	0.802	0.829	0.765
Decision tree	0.820	[0.740–0.851]	0.802	0.751	0.718	0.737
KNN	0.821	[0.737–0.853]	0.810	0.803	0.823	0.761

RF, random forest; SVM, support vector machine; KNN, K-nearest neighbors

On the other hand, the baseline model development utilizing the MIMIC-III Cohort, RF proved to be the best baseline model in both cases, that we used tabular format of data or the transformed data as inputs. In the case that we used the tabular format of the data as inputs RF resulted in the following metrics, AUROC = 0.713, 95% CI = [0.691–0.761], precision = 0.750, sensitivity = 0.801, accuracy = 0.826, and F-score = 0.760. Also, in the case that we used TSS, demographics, and the severity scores as inputs, RF resulted in the following metrics, AUROC = 0.841, 95% CI = [0.793–0.864], precision = 0.820, sensitivity = 0.803, accuracy = 0.826, and F-score = 0.771. It can be observed that the results of the proposed approach are far better than the results of the best baseline model.

In terms of model cost, all the tests were performed on a computer running Windows 10 with an Intel i7-6700 CPU and 16 GB RAM. Also, the associated computational times to the different steps of the proposed methods were as follows: Conversion of EHR to the event logs = 55 min, generating a process model through process mining discovery algorithm = 15 min, producing Timed State Sample thorough DREAM algorithm = 20 min, training the model = 60 min, and testing the model took less than 30 s.

### Shapley value analysis

**Fig. 3:**
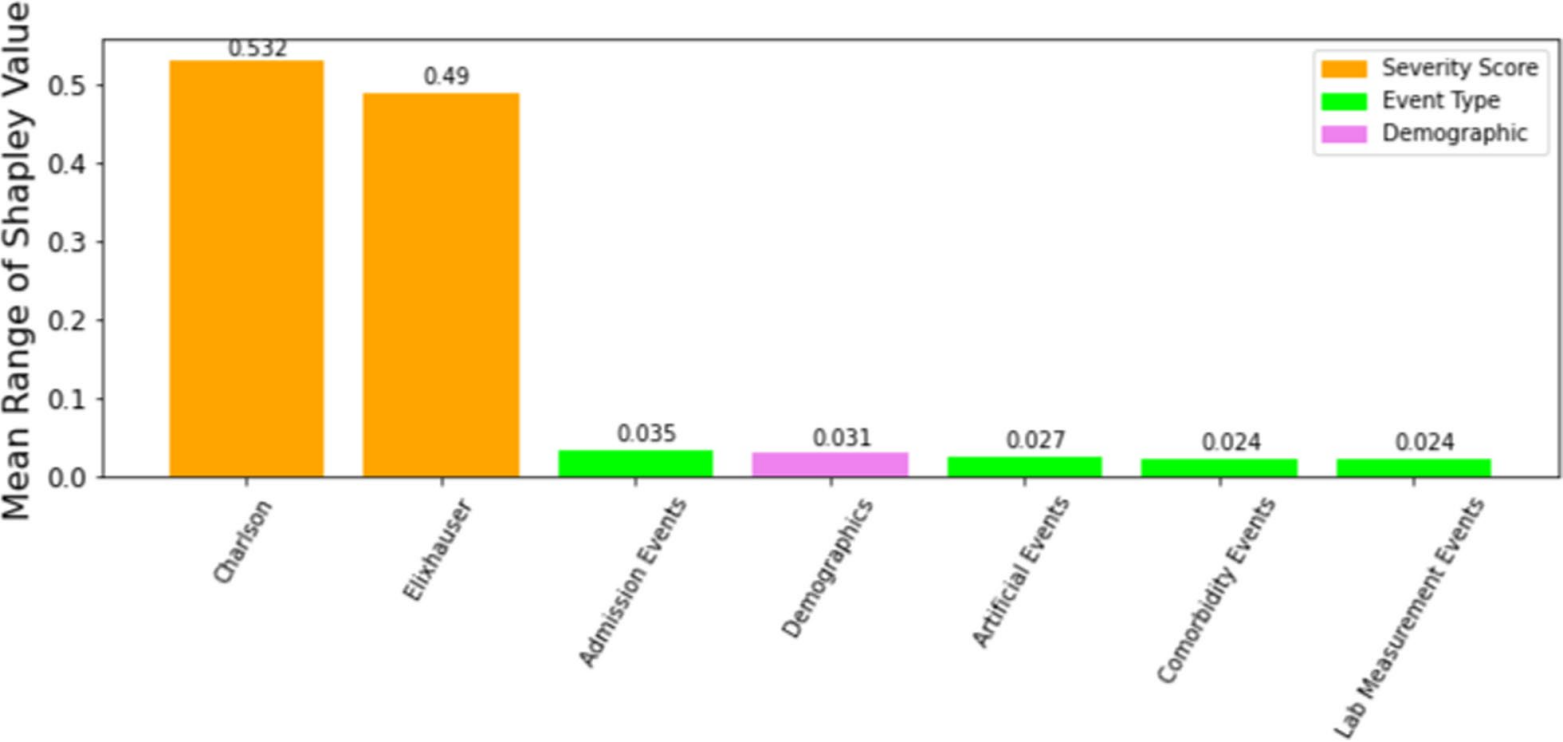
The Mean range of Shapley Values for each variable type. This Figure illustrates the impact of each variable in predicting unplanned 30-day readmission of ICU HF patients. The severity scores (Charlson and Elixhauser) have the highest impact in prediction. Following the severity scores, admission events, demographics, artificial events, comorbidity events and lab measurements events have some impact in prediction in order

Figure 3 illustrates the results of the Shapley value analysis. Based on this figure, severity scores had the most significant impact on the prediction of unplanned 30-day readmission of the HF ICU patients, followed by admission events and demographic information that seemed to have a similar impact on prediction. Whereas artificial events, comorbidity events, and lab measurement events were the least important variables for the prediction of the outcome in order. Among the severity scores, Charlson had a higher impact on prediction compared to that of Elixhauser which showed that the severity of the conditions played an important role in the prediction of unplanned 30-day readmission of ICU HF patients since Charlson score assigns higher weights to the severity level of the conditions than Elixhauser.

The Shapley value analysis confirmed that the severity scores had the highest impact on prediction in our model. However, other contributing factors impacted the prediction of the outcome including admission events, demographics, artificial events, comorbidity events, and lab measurement events which were all ignored by health calculators as the inputs for prediction.

### Variable ablation study

**Fig. 4:**
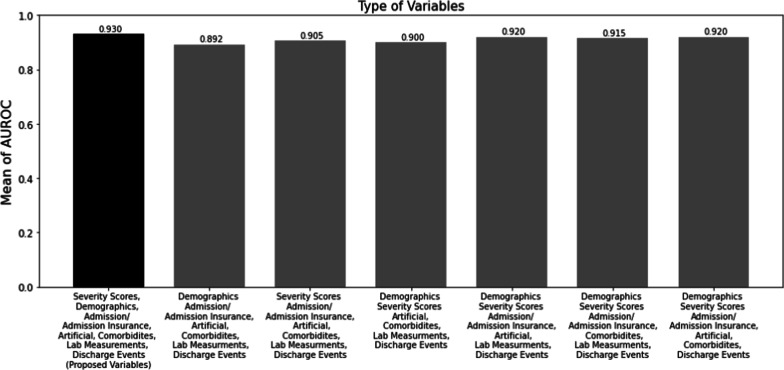
Ablation study on the variable types

The selection of the variables initially was done based on the existing literature and the experts’ opinions which was explained in detail in the variable selection subsection. Moreover, variable ablation was performed to find out the importance of each selected variable. Variable ablation was performed, firstly, by removing severity scores, and the rest of the data were used as inputs to the proposed model which led to the AUROC of 0.892. In the next step, the demographic information was removed and the rest of the data were fed to the proposed model and led to the AUROC of 0.905. Moreover, the stepwise event type removal from the original event logs was done. The impact of each event type was investigated by removing specific event types from the event logs and ten times running the proposed model by using the resultant event logs along with severity scores and demographics information as inputs. The discharge event was never removed from the event logs since they were required to be marked as the state in which the Timed State Samples were extracted, and the unplanned 30-day readmission was predicted. As a result, four different event logs were built. The details of the event types in each event log, and the resultant AUROCs are shown in Fig. 4. The results in Fig. 4 indicated that removing severity scores, demographics, admission/admission insurance type, artificial events, comorbidities event, and lab measurements were led to AUROC of 0.892, 0.905, 0.900, 0.915, 0.920, and 0.920, respectively. As a result, the performance maximization was reached when severity scores, demographics, and all event types included in the event logs, were used as inputs to the proposed model.

### Neural network layer ablation study

**Fig. 5:**
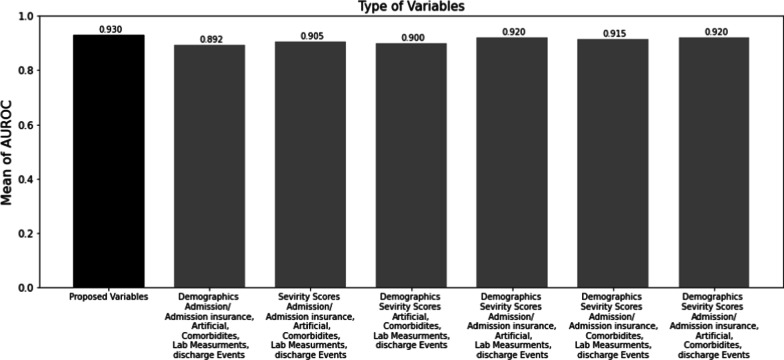
Ablation study on the variable types

The proposed NN architecture was analyzed in two steps. First, an analysis was performed by adding further layers to each of the three inputs prior to the concatenation layer. Second, a step-wise decrease of layer after the concatenation was performed. The results are visualized in Fig. 5. Figure 5 indicates that having only one layer per input prior to concatenation led to AUROC of 0.902 over ten runs, adding the second layer led to the AUROC of 0.909, adding the third layer led to the AUROC of 0.930, however, adding the fourth layer led to the AUROC of 0.920. This justified that having three layers per input prior to concatenation is the optimal number of layers. On the other hand, the removal of the post concatenation layer (except for the softmax output) led to an AUROC of 0.914. As a result, having one post concatenation layer was the optimum which led to an AUROC of 0.930. The results indicated that the proposed architecture is locally optimized.

## Discussion

### Existing model compilation summary

Several methods have been concurrently developed to predict unplanned 30-day readmission of the ICU patients with HF aiming to benefit both health care providers and the patients. Table 6 shows the existing models that have been used MIMIC-III dataset to predict unplanned 30-day readmission of ICU patients with HF.

**Table 6:** Summary of the existing models and their performance on the MIMIC-III dataset

Study	Method	Variables^a^	Performance
Hu et al. [8]	Constrained support vector machine (cSVM)	BUN, DBP, FIO, Glucose, HR, RR, SBP, Temperature, Weight, pH, FIO, HR, MBP, OS, RR, SBP, Temperature, Weight, LOS, GCS eye, GCS verbal, Age, Gender, Race, Insurance, Discharge location	AUROC 0.680 95% CI: 0.651–0.722
Baruah [19]	CNN	Clinical notes	AUROC 0.646 Precision 0.876 Sensitivity 0.697
Liu et al. [39]	Random Forest (RF), Convolutional Neural, Networks (CNN)	Clinical notes	Precision 0.698 Sensitivity 0.771 Accuracy 0.733
Huang et al. [40]	bidirectional transformer model (Clinical Bert)	Clinical notes	AUROC 0.768

BUN, blood urea nitrogen; DBP, diastolic blood pressure; FIO, fraction of inspired oxygen; HR, heart rate; RR, respiratory rate; SBP, systolic blood pressure; MBP, mean blood pressure; OS, oxygen saturation; LOS, length of stay; GCS eye, glasgow coma scale eye opening; GCS verbal, glasgow coma scale verbal response

^a^The mean and std were used for the continuous variables in Hu et al. [15] research papers

In this study, a process mining/deep learning technique was investigated for predicting unplanned 30-day readmission of ICU patients with HF, in which time information associated with the events, severity scores, and demographics were fed into a NN model.

The effectiveness of our developed approach outperformed the best results of the existing literature in terms of the AUROC value proposed by Lin et al. [11]. The efficacy of our approach was demonstrated by a substantial improvement of + 10% on AUROC.

In addition, the presented results indicated + 6% and + 7% improvements in sensitivity and F-score metrics, respectively, compared to the best sensitivity and F-score values reported in the literature by Huang et al. [39].

Although the existing proposed methodologies in the literature were successful in predicting unplanned 30-day readmission of ICU patients with HF, they possessed several drawbacks. First, most of the existing models did not use the time-series features, and to the best of our knowledge, none of them incorporated time information associated with the variables in their predictive modeling that could lead to significant information loss and poor performance accordingly [40, 41].

Furthermore, the proposed approach was a process mining/deep learning approach that illustrated the *careflows* of patients through a process model. As a result, our framework was more interpretable compared to the existing methods, which is significant for clinical applications [42].

Moreover, the health calculators that computed outcomes based on the severity scores ignored the past medical history of the patients which could have a significant impact on the likelihood of unplanned readmission.

Our proposed approach had several advantages over prior research papers which are as follows: (a) Process mining approach yielded a comprehensive analysis of *careflows* of patients through a process model which is understandable and can easily be interpreted compared to ML techniques. The process model provided a map that represented the possible diagnosis, procedures, performed services, laboratory measurements, and more, that happened to a patient. Additionally, it eased the interpretability of a model prediction. An example of a process model can be found in the existing research paper [20, 43] (b) The EHR can be directly used as inputs to our proposed approach without any computationally expensive preprocessing steps. (c) The process mining/deep learning framework was capable of modeling the time-related variables and incorporating the medical history of the patients from the previous hospital visits in the prediction algorithm unlike ML-based models and health calculators.

### Study limitations

The proposed approach had some limitations. Even though MIMIC-III is a comprehensive database and many recent research projects have been using the same database for their experiments [21, 44], the data is almost 18 years old. Thus, we suggest that a newer multihospital database such as the Nationwide Readmission Database (NRD) [23, 45] should be used in the future to externally validate our proposed model and its results. Also, MIMIC-III readmission information is limited to several facilities, and for the cases that the patients are admitted to other facilities, the readmission information is not available, hence, it may bias the results. Since this approach was a process mining/deep learning approach, the availability of the past hospital visits of the patients was essential. This approach was not useful for patients whose admission histories were not available. However, this limitation can be overcome if the history of patients could be exchanged through a network system between health care providers. Application Program Interfaces (APIs) and similar innovations hold promise that soon these drawbacks can seemingly be curtailed.

Moreover, in our model development, the train and validation datasets were used to build the model. The test dataset was set aside from the beginning and only used to evaluate the performance of the model. The train, validation, and test sets were coming from the MIMIC-III dataset. However, using an independent dataset from a different system would be beneficial to test the performance of the model [46], which provides room for future work.

## Conclusions

A process mining/deep learning approach to model EHR data of ICU patients with HF to predict unplanned 30-day readmission provided significant improvement in outcome prediction observed and compared to the results of the baseline ML models and existing literature. This improvement could be due to the capability of the process mining approach of modeling time information related to the variables and incorporating past hospital visits of the patients for prediction. Our framework can assist clinicians in identifying patients with a higher risk of unplanned 30-day readmission. Discharge planners may find this prediction tool useful in determining when a patient is safe to be discharged from the hospital and to guide post-discharge outpatient management. Future studies may validate the proposed approach using datasets from other healthcare systems or investigate its use for different diseases and outcomes. Moreover, the MIMIC-III dataset contains useful information such as clinical notes, and images, which can be fed to the models as inputs. Therefore, it potentially makes room for further research.

## Data Availability

The MIMIC-III database which was used during the current study is publicly available [25].

## Abbreviations

DREAM: Decay replay mining
NN: Neural network
TSS: Time state sample
BUN: Blood urea nitrogen
MIMIC-III: Medical Information Mart for Intensive Care III
std: Standard deviation
Pro-BNP: Pro-brain natriuretic peptide
